# Mitochondrial transfer and mesenchymal stem cells in ophthalmology: current evidence and therapeutic implications

**DOI:** 10.3389/ebm.2026.11128

**Published:** 2026-06-30

**Authors:** Xiaoli Liu, Mingqi Zhang, Zhuoshi Wang

**Affiliations:** 1 Liaoning Key Lab of Ophthalmic Stem Cells, He University, Shenyang, China; 2 School of Pharmacy, He University, Shenyang, China; 3 Science and Education Department, He Eye Specialist Hospital, Shenyang, China

**Keywords:** clinical translation, mesenchymal stem cells, mitochondrial transfer, ophthalmic diseases, retinal degeneration

## Abstract

Mitochondrial dysfunction, driven by genetic mutations or oxidative stress, is a central contributor to the onset and progression of ophthalmic diseases. In recent years, intercellular mitochondrial transfer (MT) has emerged as a novel mechanism of cellular communication and repair in ocular tissues. MT occurs through tunneling nanotubes, extracellular vesicles (EVs), cell fusion, or transmitophagy, and has been shown to support photoreceptor survival, maintain retinal homeostasis, and protect against oxidative injury. Mesenchymal stem cells (MSCs), owing to their remarkable reparative and immunomodulatory properties, have attracted particular attention as efficient mitochondrial donors. Evidence from experimental models demonstrates that MSC-mediated MT can restore bioenergetics, mitigate oxidative stress, and rescue cellular function in inherited optic neuropathies, corneal injuries, retinal degenerative diseases, and ischemic retinopathies. This review summarizes current evidence of MT in ophthalmology, highlights the therapeutic contributions of MSCs, discusses the molecular and microenvironmental factors regulating MT efficiency, and outlines unresolved challenges. We further provide perspectives on how mitochondrial transfer may be translated into innovative therapies for ocular disorders.

## Impact statement

To our knowledge, this review is one of the first comprehensive summaries focusing on mitochondrial application of mesenchymal stem cells in ophthalmology. As a novel emerging field, mitochondrial transplantation opens up a new direction for stem cell therapy, addressing the urgent need for innovative therapeutic strategies in ophthalmic diseases. It advances the field by systematically organizing the latest research findings, clarifying the potential of mesenchymal stem cell mitochondria in treating ocular disorders. The review imparts new perspectives on the clinical transformation value of this novel technology, guiding subsequent basic and clinical research, and promoting the development of innovative stem cell-based therapeutic approaches for refractory ophthalmic diseases.

## Background

Mitochondria, the “powerhouses of the cell,” generate ATP through oxidative phosphorylation and are essential for calcium signaling, reactive oxygen species (ROS) regulation, apoptosis, and inflammation [1, 2]. Balanced mitochondrial fusion, fission, biogenesis, and mitophagy preserve mitochondrial integrity and function [2]. Mutations in mitochondrial or nuclear DNA disrupt these processes, leading to structural and functional abnormalities collectively termed mitochondrial diseases [3].

The first inherited mitochondrial optic neuropathy described was Leber hereditary optic neuropathy (LHON), caused by three primary mtDNA mutations (ND1 G3460A, ND4 G11778A, and ND6 T14484C). These mutations impair complex I, reduce ATP production, elevate ROS, and selectively damage retinal ganglion cells (RGCs) and the optic nerve [4]. Mitochondrial dysfunction is also a key pathogenic driver in secondary ophthalmic disorders, such as glaucoma, diabetic retinopathy (DR), and age-related macular degeneration (AMD) [5–8].

Conventional therapies—including pharmacological agents, laser treatments, and surgery—slow progression but fail to restore function in severely damaged corneal, retinal, or optic nerve tissues [9]. Mesenchymal stem cells (MSCs) have thus gained attention for their regenerative potential in ocular diseases [10]. While MSCs exert effects through differentiation, paracrine signaling, and immune regulation, growing evidence highlights mitochondrial transfer (MT) as an additional mechanism of repair [11].

This review provides a systematic overview of MT in ocular cells, with emphasis on MSC-mediated MT (Table 1). We discuss its therapeutic applications across diverse ophthalmic diseases, analyze key regulatory factors, and propose perspectives for clinical translation.

**TABLE 1:** Application of MSCs derived mitochondria in various ophthalmic diseases.

Disease	Source	Model	Methods	Route	Action	References
LHON	Human UC-MSCs	LHON iPSC derived NPCs	Mitochondria reporter systems; Quantifying mitochondrial DNA	Not verified	Improve mitochondrial function and increase the proportion of normal mitochondrial DNA in LHON, as well as ameliorate the electrophysiological function of neurons differentiated from LHON patient-derived neural progenitor cells	[12]
LHON	Rat ASCs	LHON model cells (GM10742 cells); mtND4R340H mtTg LHON male mice; rot-induced LHON male mouse model	Mitochondria reporter systems; mitochondrial dyes	Extracellular vesicles	Restore mitochondrial function and alleviate LHON-associated symptoms in LHON male mice, as well as promote mitochondrial function in the rot-induced pan-mitochondrial disease model	[13]
Leigh syndrome	Human iPSC- MSCs	Ndufs4 gene knockout mice*; primary mouse RGCs	Mitochondria reporter systems; Quantifying mitochondrial DNA; mitochondrial dyes	Not verified	Reduce the loss of RGCs, promote mitochondrial transfer to RGCs across the ILM to protect retinal function, and inhibited abnormal activation of MG as well as the inflammatory state of the degenerated retina	[14]
Corneal injury (alkaline burns)	Human iPSC- MSCs	Primary hCECs; alkali-injured rabbit model	Mitochondria reporter systems; mitochondrial dyes	TNTs	Reduce oxidative stress-induced mitochondrial damage and rapid repair of alkaline burn induced corneal mitochondrial dysfunction	[15]
Corneal injury (acid burn)	Human UC-MSCs derived mitochondria	Primary human corneal epithelial cells; mice with acid-induced corneal burns	Mitochondria reporter systems; mitochondrial dyes	Co-culture; subconjunctival injection	Reduce apoptosis and oxidative stress levels, inhibite acid burn–induced inflammatory cell infiltration, and accelerate the repair process in a mouse model of corneal acid burn	[16]
AMD	BMMCs derived mitochondria	oAβ treated ARPE-19 cells; oAβ treated mice	Mitochondria reporter systems; mitochondrial dyes	Co-culture; intravitreal injection are transferred to the RPE layer	Enhance Aβ clearance, and prevent disruption of tight junctions	[17]
AMD	UC-MSCs derived mitochondria	Replicative senescence-induced ARPE-19 cells; Oxidative stress induced senescence in ARPE-19 cells	Mitochondria reporter systems	Co-culture	Improve mitochondrial dysfunction and alleviate cellular senescence hallmarks	[18]
AMD	Human WJ-MSCs derived mitochondria; human E-MSCs derived mitochondria	Oxidative damage (H_2_O_2_)-induced ARPE-19 cells	Mitochondrial dyes (TMRM staining)	Co-culture	WJ MSCs: Enhance cell survival and activate autophagy pathways E-MSCs: Repair retinal damage and enhance RPE function	[19]
RD	BMSCs; BMSCs derived mitochondria	Rotenone treated primary MG; RCS rats	Mitochondria reporter systems	TNTs and cell fusion	Integrated into the mitochondrial network of MG; restore mitochondrial structural and functional damage, reduce oxidative stress and gliosis, and partially protecte the visual function of degenerative rat retina	[20]
Ocular tissue regeneration	UC-MSCs	Corneal endothelial cells; 661W cells; ARPE-19 cells; rotenone treated corneal endothelial cells; C57BL/6J mice	Mitochondria reporter systems	TNTs	Increase aerobic capacity and upregulation of mitochondrial genes	[21]
Retinal degenerative diseases	Human ASCs	RPE-1 cells with hypoxia treatment or serum starvation	Mitochondrial dyes	TNTs	Support cellular stability under stress	[22]
Ischemic retinopathies	Human iPSC- MSCs; ASCs	THP-1 cells, primary mouse splenocyte; ocular hypertension-induced ischemia-reperfusion injured mice	Mitochondria reporter systems	TNTs	Reprogram mouse CD4^+^T cells into CD4^+^CD25^+^Foxp3^+^Tregs and enable Foxp3+Tregs to be recruited to counteract inflammation and retinal degeneration Induced by ischemia-reperfusion injury *in vivo*	[23]
Retinal ischemia	Rat primary BMSCs	MCAO sprague-dawley rat model and an OGD RPE cell model	Mitochondrial dyes	Not verified	Restore mitochondrial function, mitochondrial network morphology, and mitochondrial dynamics in OGD cell models, while reducing RGC loss and optic nerve damage in MCAO rats	[24]

* The NADH: ubiquinone oxidoreductase iron-sulfur protein 4 (Ndufs4) gene knockout (KO) mice, initially used as a model for Leigh syndrome, were characterized by mitochondrial complex I dysfunction.

## Inherited mitochondrial diseases

### Leber hereditary optic neuropathy (LHON)

**FIGURE 1:**
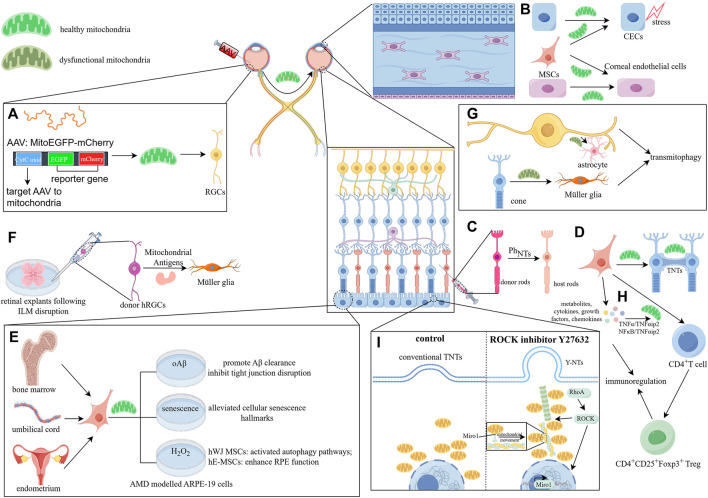
The development of mitochondrial transfer in the field of ophthalmology. **(A)** In Leber hereditary optic neuropathy (LHON) mouse models, adeno-associated virus (AAV)-loaded mitochondria travel along the visual system to the contralateral optic nerve and retina; the underlying mechanism remains unclear. **(B)** Intercellular mitochondrial transfer occurs in corneal epithelial cells (CECs) as well as between corneal endothelial cells in stress environments. Mesenchymal stem cells (MSCs), serving as efficient mitochondrial donors, participate in corneal injury repair. **(C)** Donor photoreceptors deliver proteins, RNA and functional mitochondria to host cells through photoreceptor nanotubes (^Ph^NTs). **(D)** MSCs-derived mitochondria transfer between 661W cells (a cone photoreceptor cell line) through tunneling nanotubes (TNTs). **(E)** MSCs from different sources exert mitochondrial repair effects on ARPE-19 (human RPE cell line)-based age-related macular degeneration (AMD) models. **(F)** After inner limiting membrane (ILM) disruption, transplanted human embryonic stem cell derived retinal ganglion cells (RGCs) transfer mitochondrial antigens to Müller Glia (MG), enabling MG reprogramming to supplement RGCs for cell replacement therapy. **(G)** In neurons, transmitophagy was first reported between RGC axon mitochondria and astrocytes; damaged cone mitochondria are delivered to MG for degradation under mitochondrial stress. **(H)** MSCs transfer mitochondria to T cells to suppress effector T cells and induce Treg migration and anti-inflammation. MSCs also exert immunomodulation through paracrine factors (cytokines, growth factors, EVs) and control mitochondrial transfer via the TNFα/TNFαip2 and NFκB/TNFαip2 pathway. **(I)** Proper-dose Y27632 induces longer nanotubes in ARPE-19 cells, enhancing NTs-mediated mitochondrial transport via cytoskeletal remodeling. It upregulates Miro1 to modulate mitochondrial dynamics and movement. This image is drawn by Figdraw.

LHON is the most common inherited mitochondrial optic neuropathy, with ND4 mutations accounting for most cases. Gene therapy using recombinant adeno-associated virus (AAV) vectors encoding wild-type ND4 has demonstrated significant bilateral visual improvement in clinical trials [25–30]. Interestingly, contralateral effects suggest intercellular mitochondrial or vector transfer along optic pathways (Figure 1A) [31].

Beyond gene therapy, MSC-mediated MT has emerged as a complementary strategy. Studies using induced pluripotent stem cell (iPSC)-derived models showed that MSCs could restore mitochondrial DNA ratios, improve mitochondrial function, and enhance electrophysiological activity in LHON-derived neurons [12]. In mouse models of mitochondrial complex I deficiency, intravitreal MSCs transplantation protected RGCs and preserved retinal function by delivering functional mitochondria across the inner limiting membrane (ILM) [14]. The release of mitochondria-containing EVs from MSCs also effectively delivered functional mitochondria to damaged cells, restoring mitochondrial functions in both LHON cell models and LHON-like male mice [13].

These findings indicate that MSC-mediated MT provides a promising therapeutic approach for LHON and other inherited mitochondrial disorders such as mitochondrial encephalomyopathy, lactic acidosis, and stroke-like episodes (MELAS), myoclonic epilepsy with ragged red fibers (MERRF) [32, 33].

Although gene therapy and MSC-mediated MT show promise in inherited mitochondrial optic neuropathies, several challenges remain. The mechanisms underlying the contralateral effect of lenadogene nolparvovec are still debated, and the efficiency and long-term stability of MSC-derived mitochondrial integration in human retinas are unknown. Most findings come from rodent models, raising concerns about translational relevance, while evidence from other mitochondrial diseases (e.g., MELAS, MERRF) remains preliminary.

Future studies should clarify the mechanistic basis of contralateral effects, optimize MSCs delivery to overcome retinal barriers, and establish quantitative *in vivo* tracking methods. Large-animal studies and long-term safety evaluations will be critical, and combining gene therapy with MT may ultimately offer synergistic benefits for inherited mitochondrial eye diseases.

## Corneal diseases

Corneal epithelial cells (CECs) are highly susceptible to oxidative stress and mitochondrial dysfunction, contributing to conditions such as dry eye disease and corneal chemical burns [15, 34–37]. It has been observed that intercellular MT occurs among CECs and corneal endothelial cells. Stress conditions further facilitate this process (Figure 1B), implying a damage-induced “SOS” signaling mechanism [21, 38, 39].

In alkali-burned corneal models, MSCs transplantation supported wound healing and mitochondrial repair (Figure 1B). Notably, when tunneling nanotubes (TNTs)-mediated contact was blocked, MSCs lost much of their reparative effect, confirming the essential role of MT [15]. Thus, MT complements paracrine signaling to protect corneal cells from mitochondrial dysfunction. Interestingly, human corneal epithelial cells could internalize exogenous mitochondria isolated from MSCs (Figure 1B). A single subconjunctival injection of isolated mitochondria reduced inflammatory cell infiltration induced by acid burn and accelerated the corneal repair process [16].

Most studies focus on acute injury models, and the long-term stability of transferred mitochondria in chronic corneal diseases remains unclear. Future work should evaluate chronic models, clarify the relative role of MT versus paracrine signaling, and optimize safe delivery methods for clinical translation.

## Retinal degenerative diseases

### Photoreceptor degeneration and AMD

Retinal degenerative diseases such as AMD and retinitis pigmentosa feature irreversible loss of photoreceptors and RPE cells [40]; these metabolically active, mitochondria-rich cells develop progressive mitochondrial dysfunction with reduced membrane potential, accumulated DNA mutations, increased oxidative stress and impaired quality control, which leads to energy deficiency, oxidative damage, cell death and subsequent retinal neuronal impairment [41, 42]. Photoreceptor transplantation is a potential therapy for retinal diseases, acting mainly through cell replacement and intercellular component transfer [43]. Donor photoreceptors delivered proteins, RNA and functional mitochondria to recipient cells via photoreceptor nanotubes (^Ph^NTs) [44, 45], and mitochondria derived from MSCs or donor photoreceptors can improve the metabolism of host cells (Figure 1C) [21, 44, 45]. It remains unclear how cell replacement and material transfer respectively contribute to therapeutic outcomes. Moreover, MSCs-derived mitochondria and lysosomes can also be exchanged between photoreceptors through TNTs (Figure 1D) [21].

Exogenous mitochondrial delivery from MSCs also mitigates AMD-related mitochondrial dysfunction (Figure 1E). For example, bone marrow mononuclear cells (BMMCs)–derived mitochondria improved RPE function in oligomeric amyloid beta (oAβ)–treated models [17], while umbilical cord mesenchymal stem cells (UC-MSCs) -derived mitochondria alleviated senescence-associated mitochondrial defects [18]. Tissue source influences outcomes: human Wharton’s Jelly MSCs (hWJ-MSCs) promoted autophagy, whereas human endometrium-derived MSCs(hE-MSCs) enhanced RPE function, highlighting source-dependent therapeutic mechanisms [19].

## Muller glia and transmitophagy

Müller glia (MG) play a central role in retinal homeostasis. Studies in degenerative models show that MG internalize MSCs-derived mitochondria, restoring mitochondrial structure and suppressing gliosis [20]. After ILM disruption, transplanting human embryonic stem cell derived RGCs into the mouse retina resulted in the transfer of donor RGCs substances, including mitochondrial antigens, to recipient MG (Figure 1F) [46]. Moreover, RGCs and cones can donate damaged mitochondria to glial cells for degradation, a process termed transmitophagy (Figure 1G) [47, 48]. This highlights glial cells participation in mitochondrial quality control and neuroprotection.

Although mitochondrial transfer has been implicated in photoreceptor rescue, RPE repair, and Müller glia support, several important questions remain unanswered. The relative contribution of MT versus other forms of material exchange—such as cytoplasmic or nuclear transfer—remains controversial, particularly in photoreceptor transplantation studies. Moreover, while MSCs-derived mitochondria have shown benefits in AMD and retinitis pigmentosa models, differences in therapeutic outcomes between tissue sources (e.g., Wharton’s Jelly vs. endometrium MSCs) highlight the need for systematic comparative studies. The persistence of transferred mitochondria and their functional integration into highly specialized retinal cells also remain poorly defined. Furthermore, transmitophagy appears to play a role in Müller glia support and neuroprotection, but its regulation and therapeutic implications in human disease are not well understood. Future directions should include longitudinal *in vivo* imaging, single-cell transcriptomic approaches to map donor–recipient interactions, and controlled studies comparing MT to direct mitochondrial transplantation. Ultimately, clarifying these mechanisms will be essential to translate MT into durable therapies for retinal degenerative diseases.

## Ischemic retinopathy and glaucoma

Neurovascular coupling in the retina depends on pericyte communication through interpericyte TNTs (IP-TNTs) [49]. In diabetic retinopathy, pericyte loss and disrupted TNTs contribute to vascular dysfunction [50, 51]. Similarly, glaucoma is associated with impaired IP-TNTs, leading to reduced capillary diameter and blood flow [52, 53]. Whether mitochondrial transfer through IP-TNTs contributes to neurovascular regulation remains an open question.

Previous studies have shown that MSCs exert their immunomodulatory effects by releasing various mediators, including immunosuppressive molecules, growth factors, EVs, chemokines, cytokines, complement components, and various metabolites [54, 55]. MSCs-mediated MT also modulates immune responses (Figure 1H). By transferring mitochondria to T cells, MSCs promote regulatory T cell differentiation and suppress retinal inflammation in ischemia-reperfusion models (Figure 1H) [23, 56–61]. In stroke models, MSCs restored mitochondrial morphology and reduced RGCs loss [24]. These findings suggest MT as a dual mechanism: supporting vascular integrity and regulating retinal immune homeostasis.

The exact role of MT in neurovascular coupling and visual restoration is still uncertain, and current evidence is mainly from rodent models. Future efforts should employ large-animal models and real-time tracking to clarify mechanisms and assess long-term safety.

## Factors influencing mitochondrial transfer

Under physiological conditions, MT occurs occasionally at a low frequency among ocular cells. Live-cell imaging of ^Ph^NT-connected photoreceptors revealed merely two definitive MT events across 50 cell pairs during a 60-min observation period [45]. Fluorescence assays performed after 24 h of co-culture showed that the basal transfer rate was below 5% in untreated ARPE19 cells and 4% in CECs, with an even lower rate observed in corneal endothelial cells [21, 39]. The efficiency of MT is shaped by a combination of donor cell characteristics, transfer routes, recipient cell conditions, and the surrounding microenvironment. Among donor-related factors, the source of MSCs, the functional integrity of their mitochondria, and the expression of trafficking proteins such as Miro1 play decisive roles in determining transfer capacity [14, 15, 23, 62, 63]. As superior mitochondrial donors, MSCs achieve much higher transfer efficiency than native ocular cells. In co-culture, the MT rate from MSCs to corneal endothelial cells was approximately 29.42%, greatly surpassing the low spontaneous transfer level among common ocular cells [21]. With respect to transfer routes, TNTs represent the predominant mechanism, and their formation is tightly regulated by cytoskeletal remodeling and Rho GTPase signaling [64]. Pharmacological modulation, such as the use of ROCK inhibitors (e.g., Y-27632), has been shown to enhance TNTs formation and consequently increase MT efficiency (Figure 1I) [39, 65, 66]. At optimal concentrations, Y-27632 raised MT efficiency above 20% in ARPE-19 cells and increased the rate from approximately 4% to 7%–10% in CECs [39, 66]. Besides conventional TNTs, Y-27632 induced Y-NTs, an elongated subtype of intercellular nanotubes, in ARPE-19 cells and facilitated nanotube-dependent mitochondrial transport via cytoskeletal remodeling [66]. Mechanistically, it upregulated Miro1 to regulate mitochondrial dynamics; Miro1 controls mitochondrial movement by interacting with actin filaments and microtubules, thereby boosting intercellular mitochondrial transfer [2]. In addition, alternative pathways—including EVs, gap junctions, and transient cell fusion—also contribute under specific conditions [20, 67–72]. Wang, Y., et al discovered that the release of mitochondria-containing EVs from MSCs is regulated by the CD38/IP3R/Ca^2+^ pathway. Using a non-viral gene delivery vector to overexpress CD38 in MSCs, they generated “super donor” cells that produce “Super-EV-Mitochondria” with three times the mitochondrial yield and improved quality compared to normal MSC-derived EVs [13]. Recipient cell status is another critical determinant; cells experiencing mitochondrial dysfunction or oxidative stress tend to emit “rescue” signals that increase their susceptibility to accepting functional mitochondria [21, 73]. However, this improvement is limited and cannot substantially elevate the overall transfer efficiency. After hyperosmotic injury to CECs, MT increased slightly but remained markedly lower than in the Y27632 group, rendering large-scale cell repair unfeasible [39]. Additionally, recipient ocular cell type affected mitochondrial uptake efficiency; the photoreceptor cell line 661W exhibited a greater capacity to accept mitochondria from co-cultured MSCs than ARPE-19 cells [21]. Finally, the microenvironment exerts a substantial influence, as oxidative stress, hypoxia, and inflammatory signaling pathways, such as TNFα/TNFαip2, NFκB/TNFαip2, promote TNTs formation and intercellular transfer (Figure 1H) [15, 22, 63, 74, 75]. Collectively, these regulatory factors highlight potential intervention points to optimize MSC-mediated MT for therapeutic application in ophthalmic diseases.

## Discussion

Mitochondrial transfer represents a paradigm shift in the understanding of intercellular communication and tissue repair within ophthalmology. Accumulating preclinical evidence demonstrates that MSC-mediated MT can restore cellular bioenergetics, enhance resistance to oxidative stress, and protect both retinal and corneal cells from degeneration. Despite this promise, several critical challenges must be overcome before clinical application can be realized.

Unlike systemic mitochondrial therapy, intraocular mitochondrial transplantation is restricted by the blood-ocular barrier, vitreoretinal barrier and the eye’s immune-privileged microenvironment, which greatly hinder the delivery, survival and functional integration of donor mitochondria. Common routes including intravitreal, subretinal and intravenous injection also have technical defects and potential complications. In addition, non-standard isolation and storage methods reduce mitochondrial activity [41]. The long-term efficacy, biosafety and molecular mechanisms of mitochondrial transplantation in retinal repair remain unclear.

The MitoCatch system, a cell-specific mitochondrial delivery platform, effectively addressed the inadequate targeting capability and low delivery efficiency of conventional mitochondrial transplantation. Its reliability was validated in cellular models, *ex vivo* human tissues and *in vivo* animal models. Relevant studies on LHON neurons and mouse optic nerve injury models further confirmed that targeted mitochondrial transplantation reversed mitochondrial dysfunction and prevented cellular degeneration [76]. This technology provided a new direction for the treatment of neurodegenerative diseases, optic atrophy and other intractable mitochondrial disorders.

Current researches mainly verify the short-term protective effects of MSC-mediated MT via *in vitro* cell models and animal models. These simplified models cannot fully simulate the complex *in vivo* ocular microenvironment. Moreover, there is a lack of mature quantitative methods to assess MT efficiency and functional recovery *in vivo*. Existing studies have only observed phenotypic improvements such as improved energy metabolism and relieved oxidative stress, while the regulatory roles of MT in ROS signaling, mitochondrial quality control and cell apoptosis remain poorly understood.

MSCs have been proven superior to ocular cells as mitochondrial donors. Nevertheless, the field still lacks unified criteria for donor screening and tissue-specific delivery schemes. Inconsistent donor sources and immature delivery systems may trigger abnormal cell proliferation and oncogenic risks. Comparative studies between MSC-mediated indirect mitochondrial transplantation and direct mitochondrial transplantation are required to evaluate their operability, efficacy and safety. Systematic assessments of long-term mitochondrial stability, immune responses and therapeutic outcomes in classic models such as AMD and LHON are also urgently needed.

Future studies should first establish standardized quantitative assays to dynamically monitor the *in vivo* behavior of mitochondria and evaluate therapeutic outcomes. Second, in-depth investigations are required to clarify the regulatory mechanisms of core signaling pathways including PTEN/PI3K/AKT during treatment [77]. Furthermore, given the substantial pathological heterogeneity among various retinal degenerative diseases, individualized transplantation protocols should be formulated according to disease categories and patient-specific conditions.

In summary, MT is a promising therapeutic strategy for retinal degenerative diseases. By correcting mitochondrial dysfunction, it restores cellular energy balance and mitigates oxidative damage. With continuous technological optimization, mechanistic research and safety verification, MT is expected to evolve into a mature clinical therapy for irreversible visual impairment.
